# Semi-Automatic Synthesis, Antiproliferative Activity and DNA-Binding Properties of New Netropsin and bis-Netropsin Analogues

**DOI:** 10.3390/molecules190811300

**Published:** 2014-07-31

**Authors:** Jakub Szerszenowicz, Danuta Drozdowska

**Affiliations:** 1 Saint Brunon Farmacy, Bohaterów Westerplatte 4 Str., 11-500 Giżycko, Poland; E-Mail: jakubszerszenowicz@o2.pl; 2 Department of Organic Chemistry, Medical University, Mickiewicza 2A Str., 15-222 Białystok, Poland

**Keywords:** combinatorial chemistry, solid phase synthesis, netropsin analogue, non-intercalative DNA binding agent, DNA minor groove binder

## Abstract

A general route for the semi-automatic synthesis of some new potential minor groove binders was established. Six four-numbered sub-libraries of new netropsin and bis-netropsin analogues have been synthesized using a Syncore Reactor. The structures of the all new substances prepared in this investigation were fully characterized by NMR (^1^H, ^13^C), HPLC and LC-MS. The antiproliferative activity of the obtained compounds was tested on MCF-7 breast cancer cells. The ethidium displacement assay using pBR322 confirmed the DNA-binding properties of the new analogues of netropsin and bis-netropsin.

## 1. Introduction

**Figure 1:**
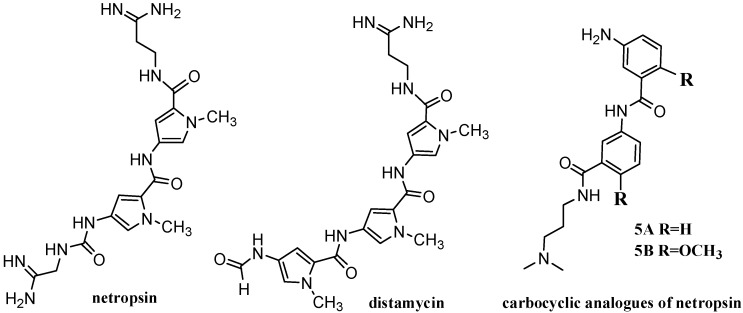
Structures of netropsin, distamycin and carbocyclic analogues of netropsin.

The polyamide antibiotics netropsin and distamycin (Figure 1), synthesized by *Streptomyces* are the most studied representatives of a series natural products possessing antitumor and antiviral properties [1]. These compounds are also well-known for their strong affinity for DNA. They bind within the minor groove of B-DNA at sites consisting of four or five consecutive AT base pairs [2]. Using netropsin and distamycin as paradigms, numerous minor groove binders have been designed and synthesized [3,4]. Analogues of these natural antibiotics, as well as other minor groove binders, have found substantial applications in anti-cancer therapy. Compounds with antibacterial, antifungal, antiviral, and antiparasitic activity have also been identified. Moreover, their importance in anti-infective therapy also has been significant and its importance is growing [5].

Distamycin and its analogues have been investigated and described very well [3,4,5,6], but analogues of netropsin have been synthesized less frequently. In the course of our investigation of minor-groove-binding drugs some netropsin analogues were synthesized and tested for DNA-binding and anticancer properties (Figure 1) [7]. It has been previously shown that those compounds inhibited proliferation of the standard MCF-7 mammalian tumor cell line with IC_50_ values of 24.43 (**5A**) and 40.73 µM (**5B**) and act as reversible minor-groove binders with selectivity for AT regions [8]. Dibenzene netropsin analogues **5A** and **5B** had not inhibited DNA topoisomerases, but they demonstrated higher DNA binding affinity in comparison to tribenzene distamycin analogues [9]. A molecular mechanics and molecular dynamics approach was used to examine the structure of the complexes formed between the d(CGCGAATTCGCG)_2_ duplex and carbocyclic analogues **5A** and **5B**. It could be expected that these compounds would be effectively isohelical with the DNA minor groove. From the analysis of our model it appears that van der Waals and electrostatic interactions are more important in stabilizing the complexes than specific hydrogen bond formation. These results were confirmed by the determination of the association constants of ligands with different polynucleotides. Compounds **5A** and **5B** bind to AT sequences with high sequence-selectivity. Moreover, the high apparent binding constants for T4 coliphage DNA gave evidence of their minor-groove selectivity [9]. The compounds of this type, containing terminal free amine groups, could be used as vectors for delivery of the DNA interacting agents. That was illustrated by the syntheses of carbocyclic analogues of netropsin with a chlorambucil moiety [10]. The syntheses of carbocyclic analogues with structures related to bis-netropsin were also described [11].

The syntheses were planned based on a previously developed new solid phase synthesis of distamycin analogues [12]. This simple and general procedure makes it possible to simultaneously carry out syntheses of many new compounds, permitting automation of the process. In the present paper results of our studies of DNA ligands as potential anticancer drugs and combinatorial synthesis of new 24-membered netropsin and bis-netropsin analogues library using a Syncore Reactor are reported. In the same study the antiproliferative activity of compounds **1**–**24** on MCF-7 breast cancer cells has also been investigated. An ethidium bromide assay was used to show that these compounds bind to plasmid pBR322.

## 2. Results and Discussion

### 2.1. Preparation of Netropsin Analogues

**Figure 2:**
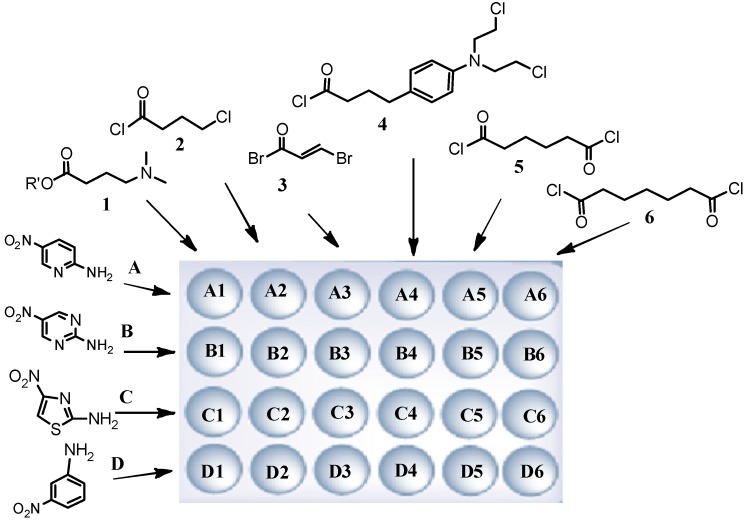
Structures of substrates and the synthesis schedule.

**Scheme 1:**
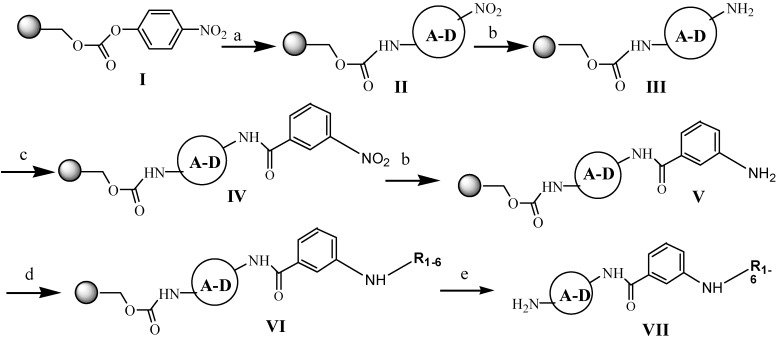
General procedure of netropsin analogues syntheses.

For the netropsin derivative preparation procedure aromatic amino-nitro compounds **A**–**D**, active ester **1** and selected acid chlorides **2**–**6**, were used as substrates to obtain four six-membered sub- libraries as shown in Figure 2. Compounds having the structure **II** were obtained according the reported procedure [12] from *p*-nitrophenyl carbonate Wang resin **I**, as shown in Scheme 1. After grafting of nitroamines to the resin, reduction of the nitro group of structure **II** was carried out using tin (II) chloride dihydrate in DMF.

Acylation of four resin-bound amines **III**, using 3-nitrobenzoyl chloride in the presence of DMAP in methylene chloride at room temperature produced the resin-bound nitro compounds with structure **IV**. Repeated reduction of the nitro groups led to compounds **V**. The next steps were reactions leading to obtaining products with structures **VI**, the final resin-bound analogues of netropsin with different moieties **R_1–6_**.

*Reagents and conditions*: (a) Aromatic nitroamine A-D, 12 h, DCM, rt; (b) 1 M SnCl_2_, 4 h DMF, rt; (c) 3-nitrobenzoyl chloride, 4-DMAP, DCM, 24 h, rt; (d) 1–6, DCM, 2 h 0 °C and rt, overnight; (e) TFA/DCM (95:5), 2 h, rt.

To combine fragments **A**-**D** with **R_1_,** the “active ester” of 4-dimethylaminobutyric acid was prepared, as presented earlier [12]. Compounds with **R_2_** were prepared through acylation reactions with 4-chlorobutyryl chloride. To join fragments **R_3_** and **R_4_** the chlorides of bromoacrylic acid and chlorambucil were synthesized, respectively. To synthesise compounds containing linkers **R_5_** and **R_6_**; adipoyl and pimeloyl dichlorides were used. Cleavage by 95% trifluoroacetic acid in dichloromethane gave the desired compounds having the structure **VII** in satisfactory yield. The structures, analytical and spectrometric data are presented in Table 1.

**Table 1:** Analytical and spectral data of the synthesized compounds. 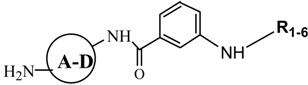

No.	Library No.	Substrate A-D	Fragment R_1_-R_6_	Purity [%]	R_t_	Formula (Exact Mass)	[M+H]^+^
**1**	**1**	**A**	**1**	96	21.98	C_18_H_23_N_5_O_2_ (341.4)	340.4
**2**		**B**	**1**	98	20.37	C_17_H_22_N_6_O_2_ (342.4)	341.40
**3**		**C**	**1**	98	20.34	C_16_H_21_N_5_O_2_S (347.4)	346.4
**4**		**D**	**1**	94	20.35	C_19_H_24_N_4_O_2_ (340.4)	339.4
**5**	**2**	**A**	**2**	87	19.03	C_16_H_17_ClN_4_O_2_ (332.8)	331.8
**6**		**B**	**2**	89	19.04	C_15_H_16_ClN_5_O_2_ (333.8)	332.8
**7**		**C**	**2**	91	19.01	C_14_H_15_ClN_4_O_2_S (338.8)	337.8
**8**		**D**	**2**	89	19.10	C_17_H_18_ClN_3_O_2_ (331.8)	330.8
**9**	**3**	**A**	**3**	98	10.27	C_15_H_13_BrN_4_O_2_ (361.2)	360.2
**10**		**B**	**3**	97	10.74	C_14_H_12_BrN_5_O_2_ (362.2)	365.2
**11**		**C**	**3**	95	10.95	C_13_H_11_BrN_4_O_2_S (367.2)	366.2
**12**		**D**	**3**	96	10.91	C_16_H_14_BrN_3_O_2_ (360.2)	359.2
**13**	**4**	**A**	**4**	97	15.89	C_26_H_29_Cl_2_N_5_O_2_ (514.4)	513.4
**14**		**B**	**4**	93	15.98	C_25_H_28_Cl_2_N_6_O_2_ (515.4)	514.4
**15**		**C**	**4**	96	15.06	C_24_H_27_Cl_2_N_5_O_2_S (520.5)	519.5
**16**		**D**	**4**	97	15.34	C_27_H_30_Cl_2_N_4_O_2_ (513.4)	512.4
**17**	**5**	**A**	**5**	99	18.80	C_30_H_30_N_8_O_4_ (566.6)	565.6
**18**		**B**	**5**	98	18.56	C_28_H_28_N_10_O_4_ (568.6)	567.6
**19**		**C**	**5**	99	18.38	C_26_H_26_N_8_O_4_S (578.7)	577.7
**20**		**D**	**5**	97	18.42	C_32_H_32_N_6_O_4_ (564.6)	563.6
**21**	**6**	**A**	**6**	99	19.98	C_31_H_32_N_8_O_4_ (580.6)	579.6
**22**		**B**	**6**	99	19.37	C_29_H_30_N_10_O_4_ (582.6)	581.6
**23**		**C**	**6**	98	19.34	C_27_H_28_N_8_O_4_S_2_ (592.7)	591.7
**24**		**D**	**6**	99	19.35	C_33_H_34_N_6_O_4_ (578.7)	577.7

### 2.2. Antiproliferative Activity of Compounds **1**–**24**

The antiproliferative effects of compounds **1***–***24** in the standard cell line of human breast cancer MCF-7 are presented as IC_50_ values in Table 2.

**Table 2:** Antiproliferative activity of compounds **1***–***24** against MCF-7 breast cancer cells.

IC_50_^a^	1	2	3	4	5	6
A	1A	2A	3A	4A	5A	6A
	74.71 µM	82.35 µM	75.511 µM	62.73 µM	234.89 µM	97.82 µM
B	1B	2B	3B	4B	5B	6B
	106.42 µM	75.34 µM	85.08 µM	86.28 µM	153.63 µM	96.48 µM
C	1C	2C	3C	4C	5C	6C
	78.35 µM	69.99 µM	71.74 µM	69.59 µM	5160.73 µM	70.01 µM
D	1D	2D	3D	4D	5D	6D
	82.31 µM	73.28 µM	70.36 µM	85.27 µM	140.74 µM	93.72 µM

^a^ The results represent the mean (±S.D.) of three independent experiments done in duplicates.

**Figure 3:**
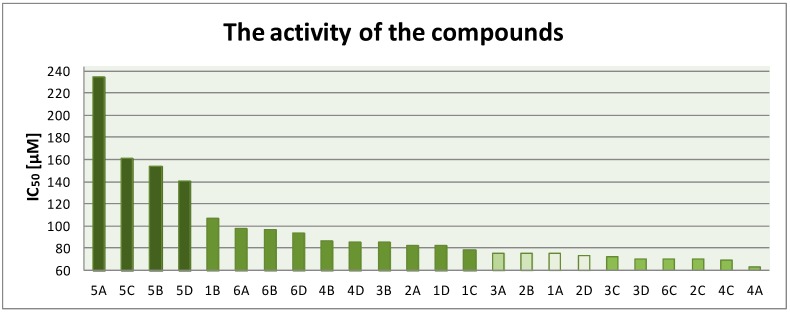
The IC_50_ values of compounds**1**–**24**.

All the tested compounds showed concentration-dependent activity, but none of the compounds were more active than netropsin with IC_50_ = 5.40 µM [11]. The concentration of the compounds that inhibited 50% of colony formation was within the range from 62.73 µM to 234.89 µM. Figure 3 shows the graph illustrating the compounds **1**–**24** in order of decreasing IC_50_ values, that is, according to their increasing antiproliferative activity.

**Figure 4:**
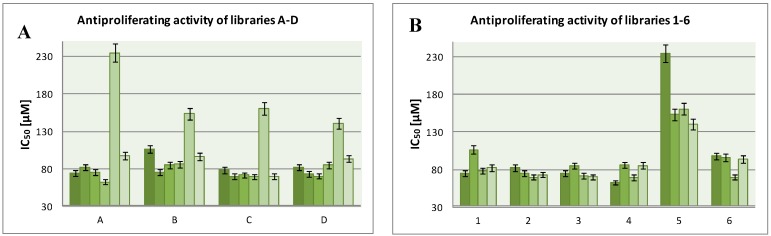
The IC_50_ values of libraries **A**–**D** (**A**) and **1**–**6** (**B**).

In order to define structure-activity relationships values of IC_50_ in particular groups of compounds: groups with different fragments **A**–**D** (Figure 4A) and libraries **1**–**6** (Figure 4B) were compared.

The above data prove that the most active of the compounds is **4A** with IC_50_ = 62.73 µM and containing a chlorambucil fragment. The least active compounds are in group **5** with (CH_2_)_4_ linkers. Higher activity of bis-netropsin analogues from library **6**, containing linker a (CH_2_)_5_ in their structures, in comparison with compounds from library **1**, was not observed. Doubling the length of the molecules also did not result in any increase of their antiproliferative activity. The compounds from libraries **2**, **3** and **4** are built with netropsin-like and alkylating fragments. Only a slight increase of activity of these compounds in comparison to netropsin analogues from library **1** was observed.

### 2.3. DNA-Binding Effects

The ethidium bromide assay showed that the investigated compounds can bind to plasmid DNA (Table 3).

**Table 3:** DNA-binding effect of compounds **1**–**24**.

	1	2	3	4	5	6
**A**	**1A** 85.63%	**2A** 98.85%	**3A** 98.80%	**4A** 70.67%	**5A** 87.27%	**6A** 84.67%
**B**	**1B** 85.45%	**2B** 98.95%	**3B** 98.90%	**4B** 74.67%	**5B** 76.36%	**6B** 69.36%
**C**	**1C** 77.73%	**2C** 79.33%	**3C** 97.33%	**4C** 85.33%	**5C** 90.67%	**6C** 78.18%
**D**	**1D** 83.18%	**2D** 86.67%	**3D** 88.00%	**4D** 64.67%	**5D** 71.81%	**6D** 69.33%

**Figure 5:**
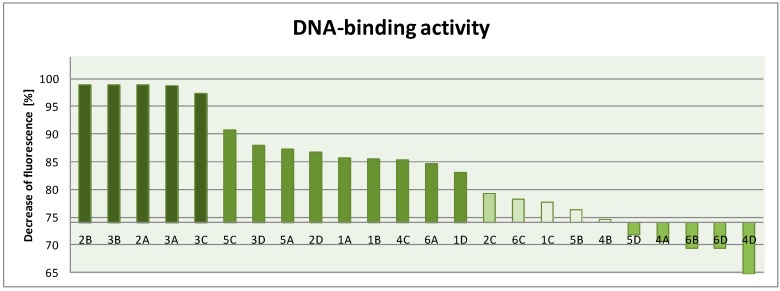
NA-binding activity of compounds **1**–**24**.

Activity of netropsin in this assay is 74.01% [11]. Some of our new compounds were characterized by a higher binding strength to pBR322 plasmid. This can be seen on the graph presented in Figure 5.

**Figure 6:**
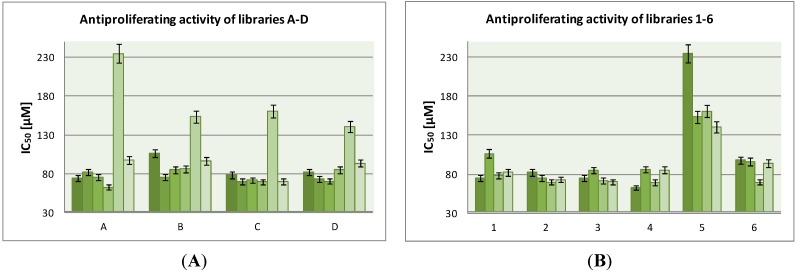
DNA-binding activity of libraries **A**–**D** (**A**) and **1**–**6** (**B**).

DNA-binding values in particular groups of compounds are shown in the Figure 6: groups with different fragments **A**–**D** (Figure 6A) and libraries **1**–**6** (Figure 6B).

Compounds **4D**, **6D**, **6B**, **4A** and **4B** bind stronger or similarly to netropsin. Three of them represent library **4**, amongst which compound **4A** is the most active against MCF-7 breast cancer cells. These data suggest that the chlorambucil fragment has been the most beneficial one in our work. It can be assumed that presence of this alkylating fragment causes additional interactions with DNA. No clear relationship between the fragments **A**–**D** and DNA binding activity was established. To confirm the binding of the investigated compounds into the minor groove requires additional research, e.g. NMR or crystallography.

## 3. Experimental Section

### 3.1 General Information

All reagents were purchased from Lancaster (Frankfurt am Main, Germany), Fluka (Sigma-Aldrich sp. z o.o., Poznań, Poland), Merck (Darmstadt, Germany), Alfa Aesar (Karlsruhe, Germany) or Iris Biotech GmBH (Marktredwitz, Germany) and used without further purification. 4-(4,6-Dimethoxy-1,3,5-triazin-2-yl)-4-methylmorpholinium tetrafluoroborate was prepared in Institute of Organic Chemistry Technical University in Łódź (Poland). Dichloromethane (DCM) and dimethylformamide (DMF) were stored under 4 Å molecular sieves. ^1^H-NMR and ^13^C-NMR spectra were recorded on a Bruker AC 400F spectrometer (Bruker corp., Fällanden, Switzerland) using TMS as internal standard; chemical shifts δ are reported in ppm. The spectra were recorded at room temperature. LC-MS spectra were recorded on Bruker Daltonics Esquire 6000 instrument with electrospray ionization (ESI). A Shimadzu LC-10A system (Shimadzu corp., Kyoto, Japan) was used for analytical HPLC (Phenomenex C18, Jupiter 90A, 4 micron, 250 × 10 mm; Phenomenex C18, Jupiter 300A, 5 micron, 250 × 4 mm; solvents: A, 0.1% aqueous TFA; B, 0.1% TFA in acetonitrile, gradient 0% B to 60% B in A in 30 min, flow rate 1 mL/min, monitored at 220 nm). Ethidium bromide was purchased from Carl Roth GmbH (Karlsruhe, Germany). Stock cultures of MCF-7 were purchased from the American Type Culture Collection (Rockville, MD, USA). Dulbecco’s modified Eagle’s medium, fetal bovine serum (FBS), netropsin, streptomycin and penicillin were products of Sigma. Plasmid pBR322 was purchased from Fermentas Life Science (Vilnius, Lithuania).

### 3.2. General Procedure

The solid-phase syntheses of the new compounds are shown in Scheme 1. 4-Nitrophenyl Wang resin **1** (0.5 g; 0.41 mmol; 0.81 mmol/g) was suspended and swelled for 10 min in dry DCM (10 mL), then the resin was treated with nitroamines (**A**, **B**, **C**, **D**; 1.64 mmol; 4 eq) dissolved in DCM (20 mL) and pyridine (177.22 μL; 2.2 mmol). [**A** (0.226 g); **B** (0.225 g); **C** (0.235 g); **D** (0.226 g)] Then the mixture was stirred for 20 h. The products **II** were washed five times with DCM (20 mL) and three times with DMF (20 mL). Then reduction of nitro groups of **II** with the dihydrate of tin (II) chloride in DMF (1 M; 20 mL) during 4 h at room temperature were carried out. The resins **III** were filtered off and then washed five times with DMF (20 mL) and three times with DCM (20 mL). Then the aliquots of 3-nitrobenzoyl chloride (0.304 g; 1.64 mmol; 4 eq) with DMAP (0.0025 g; 0.0205 mmol) were dissolved in DCM (20 mL) and added to resin **III**. The stirring was continued at room temperature overnight. After that, the resin-bound nitro compounds **IV** were washing five times with DCM (20 mL) and three times with DMF (20 mL). The treatment of thes compounds **IV** with 1 M SnCl_2_ allowed to obtain the intermediates **V**. To prepare the resin-bond compounds **VI** there were used the substrates prepared as described below. The resins **VII** were washed with DCM (5 × 20 mL), DMF (5 × 20 mL) and DCM (5 × 20 mL), dried and then treated with TFA/DCM (95:5). Subsequent evaporation of the solvent and the several portions of DCM yielded the products as glaze solids.

#### 3.2.1. Library **1**

To introduce the 4-(dimethylamino)butanamido- fragment, the “superactive ester” **1** was prepared as described earlier [12]. 4-Dimethylaminobutyric acid (0.137 g; 0.82 mmol) and NMM (902 μL; 0.82 mmol) were added to a vigorously stirred solution of 4-(4,6-dimethoxy-1,3,5-triazin-2-yl)-4-methylmorpholinium tetrafluoroborate (0.269 g; 0.82 mmol) in DMF (20 mL) and cooled to 0 °C. The stirring was continued for an additional 2 h in 0 °C, after which time these mixtures were added to the resins **V** and stirred together for an additional 2h at 0 °C and overnight at room temperature.

*N-(6-Aminopyridin-3-yl)-3-(4-(dimethylamino)butanamido)benzamide* (**A1**). Yield: 0.050 g (36%). ^1^H-NMR (DMSO-*d*_6_) δ = 10.10 (s,1H, NH), 7.22 (t,1H, NH), 6.80–7.70 (m, 7H, Ar-H), 3.95 (s, 2H, NH_2_), 2.99 (t, 2H, CH_2_), 2.72 (s, 6H, CH_3_), 2.32 (t, 2H, CH_2_); 1.85–1.88 (m, 2H, CH_2_). ^13^C-NMR (DMSO-*d*_6_) δ = 173.45 (CONH), 166.93 (CONH), 159.90 (C), 157.89 (C), 149.90 (CH), 139.72(C), 134.58 (CH), 131.85 (C), 129.61 (CH), 123.36 (CH), 119.71 (CH), 117.02 (CH), 115.64 (CH), 59.87 (CH_2_), 42.05 (CH_3_), 30.46 (CH_2_), 19.70 (CH_2_).

*N-(2-Aminopyrimidin-5-yl)-3-(4-(dimethylamino)butanamido)benzamide* (**B1**). Yield: 0.045 g (32%). ^1^H-NMR (DMSO-*d*_6_) δ = 9.48 (s,1H, NH), 7.27 (t,1H, NH), 6.85–7.35 (m, 6H, Ar-H), 3.30–4.10 (bs, 2H, NH_2_), 2.99 (t, 2H, CH_2_), 2.77 (s, 6H, CH_3_), 2.31 (t, 2H, CH_2_); 1.78–1.88 (m, 2H, CH_2_). ^13^C-NMR (DMSO-*d*_6_) δ = 173.38 (CONH), 167.48 (CONH), 159.20 (C), 157. 96 (C), 139.72(C), 134.55 (CH), 134.33 (CH), 131.52 (C), 129.10 (CH), 119.71 (CH), 116.98 (CH), 115.63 (CH), 59.85 (CH_2_), 42.23 (CH_3_), 30.35 (CH_2_), 19.36 (CH_2_).

*N-(2-Aminothiazol-5-yl)-3-(4-(dimethylamino)butanamido)benzamide* (**C1**). Yield: 0.040 g (28%). ^1^H-NMR (DMSO-*d*_6_) δ = 9.48 (s,1H, NH), 7.95 (t,1H, NH), 6.83–7.37 (m, 5H, Ar-H), 3.94 (s, 2H, NH_2_), 2.99 (t, 2H, CH_2_), 2.77 (s, 6H, CH_3_), 2.28 (t, 2H, CH_2_); 1.80–1.86 (m, 2H, CH_2_). ^13^C-NMR (DMSO-*d*_6_) δ = 173.44 (CONH), 167.50 (CONH), 162.30 (C), 157.85 (C), 134.58 (C), 131.53 (C), 129.16 (CH), 120.04 (CH), 119.29 (CH), 116.49 (CH), 115.63 (CH), 59.87 (CH_2_), 42.22 (CH_3_), 30.36 (CH_2_), 19.37 (CH_2_).

*N-(3-Aminophenyl)-3-(4-(dimethylamino)butanamido)benzamide* (**D1**). Yield: 0.049 g (35%). ^1^H-NMR (DMSO-*d*_6_) δ = 9.48 (s,1H, NH), 7.22 (t,1H, NH), 6.84–7.95 (m, 8H, Ar-H), 4.54 (s, 2H, NH_2_), 2.99 (t, 2H, CH_2_), 2.73 (s, 6H, CH_3_), 2.31 (t, 2H, CH_2_); 1.85–1.88 (m, 2H, CH_2_). ^13^C-NMR (DMSO-*d*_6_) δ = 173.45 (CONH), 166.93 (CONH), 159.90 (C), 157.89 (C), 149.90 (CH), 139.72(C), 134.58 (CH), 131.85 (C), 129.61 (CH), 123.36 (CH), 119.71 (CH), 117.02 (CH), 115.64 (CH), 59.87 (CH_2_), 42.05 (CH_3_), 30.46 (CH_2_), 19.70 (CH_2_).

#### 3.2.2. Library **2**

4-Chlorobutanamido derivatives were obtained by direct acylation of resins **V** by 4-chlorobutanoyl chloride (**2**, 2 mmol; 0.282 g; 0.224 mL). The above acid chloride was dissolved in DCM (20 mL), chilled to 0 °C and every portion was added to chilled resins **V** with DMAP (0.0025 g; 0.0205 mmol). These mixtures were stirred for 2 h in 0 °C and overnight at room temperature.

*N-(6-Aminopyridin-3-yl)-3-(4-chlorobutanamido)benzamide* (**A2**). Yield: 0.037 g (27%). ^1^H-NMR (DMSO-*d*_6_) δ = 10.08 (s,1H, NH), 7.40 (t,1H, NH), 6.88–8.22 (m, 7H, Ar-H), 3.90–4.40 (bs, 2H, NH_2_), 3.70 (t, 2H, CH_2_), 2.36 (t, 2H, CH_2_); 1.88–1.98 (m, 2H, CH_2_). ^13^C-NMR (DMSO-*d*_6_) δ = 170.43 (CONH), 167.13 (CONH), 162.29 (CH), 148.79 (C), 139.37 (C), 131.30 (C), 131.23 (CH), 131.23 (C), 128.90 (CH), 123.13 (CH), 117.93 (CH), 116.59 (CH), 114.41 (CH), 45.01 (CH_2_), 33.35 (CH_2_), 27.83 (CH_2_).

*N-(2-Aminopyrimidin-5-yl)-3-(4-chlorobutanamido)benzamide* (**B2**). Yield: 0.047 g (34%). ^1^H-NMR (DMSO-*d*_6_) δ = 10.09 (s,1H, NH), 7.21 (t,1H, NH), 6.82–8.35 (m, 6H, Ar-H), 3.30–4.10 (bs, 2H, NH_2_), 3.75 (t, 2H, CH_2_), 2.35 (t, 2H, CH_2_); 1.88–1.98 (m, 2H, CH_2_). ^13^C-NMR (DMSO-*d*_6_) δ = 171.38 (CONH), 167.79 (CONH), 159.88 (CH), 157. 96 (C), 139.72(C), 136.64 (CH), 132.24 (C), 129.38 (C), 129.18 (CH), 119.81 (CH), 117.12 (CH), 114.81 (CH), 59.85 (CH_2_), 42.23 (CH_3_), 33.37 (CH_2_), 27.86 (CH_2_).

*N-(2-Aminothiazol-5-yl)-3-(4-chlorobutanamido)benzamide* (**C2**). Yield: 0.032 g (23%). ^1^H-NMR (DMSO-*d*_6_) δ = 10.09 (s,1H, NH), 7.41 (t,1H, NH), 6.87–8.81 (m, 5H, Ar-H), 3.20–3.70 (bs, 2H, NH_2_), 3.80 (t, 2H, CH_2_), 2.35 (t, 2H, CH_2_); 1.85–1.96 (m, 2H, CH_2_). ^13^C-NMR (DMSO-*d*_6_) δ = 171.44 (CONH), 167.66 (CONH), 167.13 (C), 139.37 (C), 134.58 (C), 131.53 (C), 129.16 (CH), 120.04 (CH), 119.29 (CH), 115.34 (CH), 115.32(CH), 44.95 (CH_2_), 33.34 (CH_2_), 27.83 (CH_2_).

*N-(3-Aminophenyl)-3-(4-chlorobutanamido)benzamide* (**D2**). Yield: 0.042 g (31%). ^1^H-NMR (DMSO-*d*_6_) δ = 10.06 (s,1H, NH), 7.42 (t,1H, NH), 6.76–8.31 (m, 8H, Ar-H), 4.34 (s, 2H, NH_2_), 3.75 (t, 2H, CH_2_), 2.36 (t, 2H, CH_2_); 1.81–1.85 (m, 2H, CH_2_). ^13^C-NMR (DMSO-*d*_6_) δ = 174.35 (CONH), 167.78 (CONH), 167.12 (C), 149.90 (C), 139.72(C), 134.58 (CH), 131.31 (C), 129.61 (CH), 128.88 (CH), 123.36 (CH), 119.71 (CH), 118.09 (CH), 117.02 (CH), 115.64 (CH), 44.93 (CH_2_), 33.34 (CH_2_), 27.82 (CH_2_).

#### 3.2.3. Library **3**

Bromoacrylic acid chloride (**3**) was used to carry out the acylation reaction of resins **V**. 2-Bromoacrylic acid (2 mmol; 0.302 g) was dissolved in dry THF (20 mL), then oxalyl chloride (4 mmol; 0.508 g; 0.343 mL) was added, and warming to mild reflux under a drying tube for 2 h. The excess oxalyl chloride and solvent were removed under reduced pressure and the residue co-evaporated with dry DCM (5 mL, twice). The above acid chloride was dissolved in DCM (20 mL), chilled to 0 °C and such portions were added to chilled resins **V** with DMAP (0.0025 g; 0.0205 mmol). The mixtures were stirred for 2 h in 0 °C and overnight at room temperature.

*(E)-N-(6-Aminopyridin-3-yl)-3-(3-bromoacrylamido)benzamide* (**A3**). Yield: 0.044 g (30%). ^1^H-NMR (DMSO-*d*_6_) δ = 10.35 (s,1H, NH), 9.65 (s,1H, NH), 6.78–8.31 (m, 7H, Ar-H), 7.80 (d, 1H, CH), 7.65 (d, 1H, CH), 3.25–3.65 (bs, 2H, NH_2_). ^13^C-NMR (DMSO-*d*_6_) δ = 167.71 (CONH), 162.26 (CH), 160.74 (CONH), 148.79 (C), 139.37(C), 131.30 (C), 131.23 (C), 129.10 (CH), 128.86 (CH), 124.36 (CH), 123.20 (CH), 118.34 (CH), 117.93 (CH), 116.59 (CH), 114.41 (CH).

*(E)-N-(2-Aminopyrimidin-5-yl)-3-(3-bromoacrylamido)benzamide* (**B3**). Yield: 0.045 g (30%). ^1^H-NMR (DMSO-*d*_6_) δ = 10.36 (s,1H, NH), 9.66 (s,1H, NH), 6.62–8.31 (m, 6H, Ar-H), 7.80 (d, 1H, CH), 7.65 (d, 1H, CH), 3.20–3.45 (bs, 2H, NH_2_). ^13^C-NMR (DMSO-*d*_6_) δ = 167.78 (CONH), 162.26 (CH), 160.74 (CONH), 148.56 (C), 139.72(C), 136.64 (CH), 132.24 (C), 131.30 (C), 129.38 (CH), 128.81 (CH), 119.81 (CH), 118.05 (CH), 116.74 (CH), 114.81 (CH).

*(E)-N-(2-Aminothiazol-5-yl)-3-(3-bromoacrylamido)benzamide* (**C3**). Yield: 0.042 g (28%). ^1^H-NMR (DMSO-*d*_6_) δ = 10.87 (s,1H, NH), 9.66 (t,1H, NH), 6.80–8.31 (m, 5H, Ar-H), 7.80 (d, 1H, CH), 7.64 (d, 1H, CH), 3.30–3.70 (bs, 2H, NH_2_). ^13^C-NMR (DMSO-*d*_6_) δ = 167.75 (CONH), 162.28 (CH), 160.76 (CONH), 148.11 (C), 139.37 (C), 134.58 (C), 131.34 (C), 128.87 (CH), 120.04 (CH), 119.29 (CH), 118.33 (CH), 117.09 (CH), 114.79(CH).

*(E)-N-(3-Aminophenyl)-3-(3-bromoacrylamido)benzamide* (**D3**). Yield: 0.041 g (28%). ^1^H-NMR (DMSO-*d*_6_) δ = 10.10 (s,1H, NH), 9.12 (s,1H, NH), 6.76–8.31 (m, 8H, Ar-H), 7.80 (d, 1H, CH), 7.64 (d, 1H, CH), 3.30–3.50 (bs, 2H, NH_2_). ^13^C-NMR (DMSO-*d*_6_) δ = 167.80 (CONH), 162.30 (CH), 160.77 (CONH), 149.80 (C), 132.72 (C), 132.58 (CH), 131.32 (2C), 129.61 (CH), 128.87 (CH), 123.36 (CH), 119.71 (CH), 118.18 (CH), 116.90 (CH), 115.604 (CH), 114.64 (CH).

#### 3.2.4. Library **4**

Chlorambucil chloride (**4**) was used to carry out the acylation reaction of resins **V**. Chlorambucil (2 mmol; 0.608 g) was dissolved in dry THF (20 mL), then oxalyl chloride (4 mmol; 0.508 g; 0.343 mL) was added, and warming to mild reflux under a drying tube for 2 h. The excess oxalyl chloride and solvent were removed under reduced pressure and the residue co-evaporated with dry DCM (5 mL, twice). The above acid chloride was dissolved in DCM (20 mL), chilled to 0 °C and such portions were added to chilled resins **V** with DMAP (0.0025 g; 0.0205 mmol). The mixture were stirred for 2 h in 0 °C and overnight at room temperature.

*N-(6-Aminopyridin-3-yl)-3-(4-(4-(bis(2-chloroethyl)amino)phenyl)butanamido)benzamide* (**A4**). Yield: 0.060 g (28%). ^1^H-NMR (DMSO-*d*_6_) δ = 10.08 (s,1H, NH), 7.40 (t,1H, NH), 6.65–8.42 (m, 11H, Ar-H), 3.70–4.30 (bs, 2H, NH_2_), 3.69 (t, 8H, CH_2_), 2.47 (t, 2H, CH_2_), 2.17 (t, 2H, CH_2_), 1.65–1.88 (m, 2H, CH_2_). ^13^C-NMR (DMSO-*d*_6_) δ = 174.31 (CONH), 167.17 (CONH), 161.89 (CH), 157.10 (C), 144.46 (C), 139.52 (C), 137.91 (C), 131.35 (C), 131.23 (CH), 131.20 (C), 129.54 (CH), 129.28 (CH), 129.00 (CH), 128.84 (CH), 125.32 (C), 124.53 (C), 123.13 (CH), 123.09 (CH), 121.08 (CH), 119.81 (CH), 111.97 (CH), 52.24 (2CH_2_), 41.17 (2CH_2_), 33.60 (CH_2_), 33.08 (CH_2_), 26.57 (CH_2_).

*N-(2-Aminopyrimidin-5-yl)-3-(4-(4-(bis(2-chloroethyl)amino)phenyl)butanamido)benzamide* (**B4**). Yield: 0.056 g (26%). ^1^H-NMR (DMSO-*d*_6_) δ = 10.09 (s,1H, NH), 7.30 (t,1H, NH), 6.65–8.43 (m, 10H, Ar-H), 3.30–4.10 (bs, 2H, NH_2_), 3.70 (t, 8H, CH_2_), 2.47 (t, 2H, CH_2_), 2.19 (t, 2H, CH_2_), 1.64–1.80 (m, 2H, CH_2_). ^13^C-NMR (DMSO-*d*_6_) δ = 174.26 (CONH), 167.14 (CONH), 161.87 (CH), 157. 08 (C), 144.44 (C), 139.72 (C), 137.89 (C), 132.24 (C), 131.18 (C), 129.52 (CH), 129.26 (CH), 128.82 (CH), 125.29 (CH), 124.51 (CH), 123.11 (CH), 121.07 (CH), 119.79 (CH), 111.95 (CH), 51.99 (2CH_2_), 40.88 (2CH_2_), 33.58 (CH_2_), 33.06 (CH_2_), 26.54 (CH_2_).

*N-(2-Aminothiazol-5-yl)-3-(4-(4-(bis(2-chloroethyl)amino)phenyl)butanamido)benzamide* (**C4**). Yield: 0.061 g (29%). ^1^H-NMR (DMSO-*d*_6_) δ = 10.08 (s,1H, NH), 7.40 (t,1H, NH), 6.65–8.83 (m, 9H, Ar-H), 3.20–3.80 (bs, 2H, NH_2_), 3.65 (t, 2H, CH_2_), 2.45 (t, 2H, CH_2_), 2.18 (t, 2H, CH_2_), 1.65–1.80 (m, 2H, CH_2_). ^13^C-NMR (DMSO-*d*_6_) δ = 171.44 (CONH), 171.26 (CONH), 167.16 (C), 162.30 (C), 144.45 (C), 139.37 (C), 134.58 (C), 131.53 (C), 129.69 (CH), 129.16 (CH), 128.93 (CH), 128.84 (CH), 120.04 (CH), 119.29 (CH), 111.94 (2CH), 111.66 (CH), 52.23 (2CH_2_), 40.94 (2CH_2_), 33.33 (CH_2_), 33.07 (CH_2_), 26.56 (CH_2_).

*N-(3-Aminophenyl)-3-(4-(4-(bis(2-chloroethyl)amino)phenyl)butanamido)benzamide* (**D4**). Yield: 0.059 g (28%). ^1^H-NMR (DMSO-*d*_6_) δ = 10.06 (s,1H, NH), 7.42 (t,1H, NH), 6.64–8.24 (m, 12H, Ar-H), 4.20–4.90 (bs, 2H, NH_2_), 3.69 (t, 2H, CH_2_), 2.35 (t, 2H, CH_2_); 1.64–1.85 (m, 2H, CH_2_). ^13^C-NMR (DMSO-*d*_6_) δ = 174.32 (CONH), 171.32 (CONH), 162.30 (CH), 158.42 (C), 157.74 (C), 145.21 (C), 144.46 (C), 139.72 (C), 131.20 (C), 129.77 (CH), 129.61 (CH), 129.11 (CH), 128.86 (CH), 123.72 (CH), 123.12 (CH), 119.79 (CH), 119.16 (CH), 113.30 (CH), 111.93 (CH), 111.54 (CH), 52.22 (2CH_2_), 39.52 (2CH_2_), 33.34 (CH_2_), 33.07 (CH_2_), 26.57 (CH_2_).

#### 3.2.5. Library **5**

Bis-netropsin derivatives in library **5** were prepared by direct acylation of resins **V** by adipoyl chloride (**5**, 2 mmol; 0.366 g; 0.293 mL). The above acid chloride was dissolved in DCM (20 mL), chilled to 0 °C and this solution was added to chilled resins **V** with DMAP (0.0025 g; 0.0205 mmol). The mixtures were stirred for 2 h in 0 °C and overnight at room temperature.

*N^1^,N^6^-bis(3-((6-Aminopyridin-3-yl)carbamoyl)phenyl)adipamide* (**A5**). Yield: 0.042 g (36%). ^1^H-NMR (DMSO-*d*_6_) δ = 10.08 (s, 2H, NH), 7.62 (t, 2H, NH), 6.71–8.06 (m, 14H, Ar-H), 5.20–5.50 (bs, 4H, NH_2_), 2.37 (t, 4H, CH_2_); 1.66 (t, 4H, CH_2_). ^13^C-NMR (DMSO-*d*_6_) δ = 174.32 (2CONH), 171.29 (2CONH), 164.99 (2C), 153.67 (2C), 139.38(2C), 135.93 (2CH), 130.69 (2C), 128.58 (2CH), 122.18 (2CH), 121.72 (CH), 120.84 (2CH), 118.49 (2CH), 115.15 (2CH), 114.95 (CH), 36.25 (2CH_2_), 24.83 (2CH_2_).

*N^1^,N^6^-bis(3-((2-Aminopyrimidin-5-yl)carbamoyl)phenyl)adipamide* (**B5**). Yield: 0.047 g (40%). ^1^H-NMR (DMSO-*d*_6_) δ = 10.09 (s, 2H, NH), 7,60 (t, 2H, NH), 6.71–8.15 (m, 12H, Ar-H), 4.10–5.00 (bs, 4H, NH_2_), 2.37 (t, 4H, CH_2_); 1.66 (t, 4H, CH_2_). ^13^C-NMR (DMSO-*d*_6_) δ = 174.32 (2CONH), 171.29 (2CONH), 164.99 (2C), 153.676 (2C), 139.37 (2C), 135.93 (CH), 130.68 (2C), 128.59 (2CH), 122.17 (2CH), 121.73 (2CH), 118.49 (2CH), 115.14 (2CH), 36.26 (2CH_2_), 24.83 (2CH_2_).

*N^1^,N^6^-bis(3-((2-Aminothiazol-4-yl)carbamoyl)phenyl)adipamide* (**C5**). Yield: 0.034 g (29%). ^1^H-NMR (DMSO-*d*_6_) δ = 10.08 (s, 2H, NH), 7.60 (t, 2H, NH), 6.81–8.22 (m, 10H, Ar-H), 3.30–3.90 (bs, 4H, NH_2_), 2.36 (t, 4H, CH_2_); 1.51 (t, 4H, CH_2_). ^13^C-NMR (DMSO-*d*_6_) δ = 174.30 (2CONH), 171.30 (2CONH), 167.16 (2C), 162.30 (2C), 139.47 (2C), 131.53 (2C), 128.88 (2CH), 123.11 (2CH), 119.79 (2CH), 115.82 (2CH), 115.63 (2CH), 36.23 (2CH_2_), 24.75 (2CH_2_).

*N^1^,N^6^-bis(3-((4-Aminophenyl)carbamoyl)phenyl)adipamide* (**D5**). Yield: 0.048 g (41%). ^1^H-NMR (DMSO-*d*_6_) δ = 10.08 (s, 2H, NH), 7.60 (t, 2H, NH), 6.71–8.05 (m, 16H, Ar-H), 3.90–4.50 (bs, 4H, NH_2_), 2.37 (t, 4H, CH_2_); 1.65 (t, 4H, CH_2_). ^13^C-NMR (DMSO-*d*_6_) δ = 174.33 (2CONH), 171.29 (2CONH), 164.98 (2C), 153.66 (2C), 139.38 (2C), 135.93 (2CH), 130.68 (2C), 128.59 (2CH), 122.17 (2CH), 121.73 (2CH), 120.83 (2CH), 120.75 (2CH), 118.49 (2CH), 114.95 (2CH), 36.26 (2CH_2_), 24.82 (2CH_2_).

#### 3.2.6. Library **6**

Derivatives in library **6** were obtained using pimeloyl chloride (**6**, 2 mmol; 0.394 g; 0.327 mL), by direct acylation of resin **V**. The above acid chloride was dissolved in DCM (20 mL), chilled to 0 °C and then added to a chilled resin **V** with DMAP (0.0025 g; 0.0205 mmol). The mixtures were stirred for 2 h in 0 °C and overnight at room temperature.

*N^1^,N^7^-bis(3-((6-Aminopyridin-3-yl)carbamoyl)phenyl)heptanediamide* (**A6**). Yield: 0.045 g (38%). ^1^H-NMR (DMSO-*d*_6_) δ = 10.08 (s, 2H, NH), 7.95 (t, 2H, NH), 6.97–8.23 (m, 14H, Ar-H), 3.10–3.80 (bs, 4H, NH_2_), 2.33 (t, 4H, CH_2_); 1.48–1.56 (m, 4H, CH_2_), 1.15–1.45 (m, 2H, CH_2_). ^13^C-NMR (DMSO-*d*_6_) δ = 174.41 (2CONH), 171.46 (2CONH), 167.20 (2C), 156.96 (2C), 139.53 (2C), 139.13 (2CH), 131.24 (2C), 128.88 (2CH), 123.75 (2CH), 123.10 (4CH), 119.79 (2CH), 106.95 (2CH), 36.26 (CH_2_), 33.56 (CH_2_), 28.12 (CH_2_), 24.83 (CH_2_), 24.23 (CH_2_).

*N^1^,N^7^-bis(3-((2-Aminopyrimidin-5-yl)carbamoyl)phenyl)heptanediamide* (**B6**). Yield: 0.056 g (47%). ^1^H-NMR (DMSO-*d*_6_) δ = 10.07 (s, 2H, NH), 7,60 (t, 2H, NH), 6.95–8.23 (m, 12H, Ar-H), 3.10–3.70 (bs, 4H, NH_2_), 2.34 (t, 4H, CH_2_); 1.38–1.66 (m, 4H, CH_2_), 1.15–1.40 (m, 2H, CH_2_). ^13^C-NMR (DMSO-*d*_6_) δ = 174.40 (2CONH), 171.46 (2CONH), 167.19 (2C), 156.97 (2C), 139.37 (2C), 139.14 (2CH), 131.25 (2C), 128.88 (2CH), 123.75 (2CH), 123.11 (2CH), 119.80 (2CH), 106.96 (2CH), 36.26 (CH_2_), 33.56 (CH_2_), 28.11 (CH_2_), 24.80 (CH_2_), 24.23 (CH_2_).

*N^1^,N^7^-bis(3-((2-Aminothiazol-4-yl)carbamoyl)phenyl)heptanediamide* (**C6**). Yield: 0.052 g (43%). ^1^H-NMR (DMSO-*d*_6_) δ = 10.10 (s, 2H, NH), 7.60 (t, 2H, NH), 6.95–8.23 (m, 10H, Ar-H), 3.10–3.20 (bs, 4H, NH_2_), 2.33 (t, 4H, CH_2_); 1.40–1.70 (m, 4H, CH_2_), 1.15–1.40 (m, 2H, CH_2_). ^13^C-NMR (DMSO-*d*_6_) δ = 174.44 (2CONH), 171.52 (2CONH), 167.24 (2C), 157.01 (2C), 139.59 (2C), 139.17 (2CH), 131.59 (2C), 128.88 (2CH), 123.14 (2CH), 119.85 (2CH), 115.68 (2CH), 106.99 (2CH), 36.30 (CH_2_), 33.61 (CH_2_), 28.16 (CH_2_), 24.85 (CH_2_), 24.28 (CH_2_).

*N^1^,N^7^-bis(3-((4-Aminophenyl)carbamoyl)phenyl)heptanediamide* (**D6**). Yield: 0.044 g (37%). ^1^H-NMR (DMSO-*d*_6_) δ = 10.11 (s, 2H, NH), 7.40 (t, 2H, NH), 6.55–8.23 (m, 16H, Ar-H), 3.00–3.50 (bs, 4H, NH_2_), 2.34 (t, 4H, CH_2_); 1.40–1.70 (m, 4H, CH_2_), 1.15–1.40 (m, 2H, CH_2_). ^13^C-NMR (DMSO-*d*_6_) δ = 174.38 (2CONH), 171.46 (2CONH), 167.18 (2C), 156.94 (2C), 149.73 (2C), 139.53 (2CH), 139.07 (2CH), 131.22 (2C), 128.86 (2CH), 123.72 (2CH), 123.09 (2CH), 119.77 (2CH), 115.63 (2CH), 106.95 (2CH), 36.23 (CH_2_), 33.55 (CH_2_), 28.17 (CH_2_), 24.79 (CH_2_), 24.22 (CH_2_).

### 3.3. Pharmacology

#### 3.3.1. Cell Culture

Human breast cancer MCF-7 cells maintained in Dulbecco’s modified Eagle’s medium supplemented with 10% FBS, 50 μg/mL streptomycin, 100 U/mL penicillin at 37 °C, atmosphere containing 5% CO_2_. Cells were cultivated in Costar flasks and subconfluent cells were detached with 0.05% trypsin and 0.02% EDTA in calcium-free phosphate-buffered saline. The study was carried out using cells growing as monolayer in six-well plates (Nunc, Sarstedt, Newton, NC, USA) (5 × 10^5^ cells per well) and preincubated 24 h without phenol red.

#### 3.3.2. Determination of IC_50_ Values

The compounds were dissolved in DMSO/H_2_O (10:90) and used at concentrations of 5, 10, 15, 50 and 100 μM. Microscopic observations of cells monolayers were performed with a Nikon optiphot microscope (Nikon Instruments Inc., Melville, NY, USA). Wright-Giemsa staining was performed using the Fischer Leuko Stat Kit (Fisher Diagnostics, Orangeburg, NY, USA). After 24 h of drug treatment MCF-7 cells were mixed with a dye mixture (10 μM acridine orange and 10 μM ethidium bromide, prepared in phosphate-buffered saline). At the end of each experimental time point, all of the media was removed and cells were harvested by incubation with 0.05% trypsin and 0.02% EDTA for 1 min and washed with the medium. Then, 250 μL of cells suspension was mixed with 10 μL of the dye mix and 200 cells per sample were examined by fluorescence microscopy. There were counted percentage of non-viable (apoptotic and necrotic) cells and the concentrations which inhibited 50% of colony formation (IC_50_ values) were counted. The results were submitted to statistical analysis using the method of the smallest squares.

#### 3.3.3. Ethidium Bromide Assay

Each well of 96-well plate was loaded with Tris buffer containing ethidium bromide (0.1 M Tris, 1 M NaCl, pH 8.0, 0.5 mM EtBr final concentration, 100 μL). To each well was added 15 μg plasmid pBR322 as water solution (0.05 μg/μL). Then to each well was added netropsin or compound **1**–**24** (1 μL of a 1 mM solution in water, 10 μM final concentrations). After the incubation at 25 °C for 30 min. the. fluorescence of each well was read on a Infinite M200 fluorescence spectrophotometer (TECAN, Männedorf, Switzerland) (ex. 546 nm, em. 595 nm) in duplicate experiments with two control wells (no drug = 100% fluorescence, no DNA = 0% fluorescence). Fluorescence readings are reported as % fluorescence relative to the controls.

## 4. Conclusions

The synthesis and testing of minor groove binders analogues are the subject of constant active research. A short and efficient synthesis of 24 different netropsin analogues was developed. The presented compounds have linear chains like the natural antibiotic netropsin based on readily available building blocks, such as: pyridine, pyrimidine, thiazole and benzene rings. The replacement of N-methylpyrrole rings in netropsin by other rings yields lexitropsins with changed affinity to A-T pairs, in comparison to netropsin [11]. The introduction of the terminal 4-(dimethylamino)butanamido- fragment leads to netropsin-like compounds. Some of the new compounds (libraries **2**, **3** and **4**) have different connected alkylating groups. Compounds from libraries **5** and **6** are analogues of bis-netropsin. This protocol can be no doubt very useful in terms of speed, materials availability and generality, as well, as may provide compounds with better properties. 

The antiproliferative activity of the new compounds was determined and it was confirmed that the obtained netropsin analogues act against MCF-7 breast cancer cells, though all of them are weaker than netropsin. The ethidium displacement assay demonstrated the DNA-binding properties of the newly obtained analogues of netropsin and bis-netropsin. Five among the obtained derivatives showed stronger binding ability to DNA than the used model compound. Further investigations and testing are required to confirm whether the newly obtained compounds bind to DNA in the minor groove or in any other way.
